# Inhibitory Effect of *Bacillus licheniformis* Strains Isolated from Canine Oral Cavity

**DOI:** 10.3390/life12081238

**Published:** 2022-08-15

**Authors:** Natália Šurín Hudáková, Jana Kačírová, Miriam Sondorová, Svetlana Šelianová, Rastislav Mucha, Marián Maďar

**Affiliations:** 1 Department of Microbiology and Immunology, University of Veterinary Medicine and Pharmacy in Kosice, Komenskeho 73, 041 81 Kosice, Slovakia; 2 Clinic of Stomatology and Maxillofacial Surgery, Faculty of Medicine, University of Pavol Jozef Safarik in Kosice, 040 01 Kosice, Slovakia; 3 Institute of Neurobiology, Biomedical Research Center of the Slovak Academy of Sciences, Soltesovej 4, 040 01 Kosice, Slovakia

**Keywords:** *Bacillus licheniformis*, antimicrobial effect, oral pathogens, cell-free supernatant

## Abstract

*Bacillus licheniformis* is used in a broad spectrum of areas, including some probiotic preparations for human and veterinary health. Moreover, *B. licheniformis* strains are known producers of various bioactive substances with antimicrobial and antibiofilm effects. In searching for new potentially beneficial bacteria for oral health, the inhibitory effect of *B. licheniformis* strains isolated from canine dental biofilm against pathogenic oral bacteria was evaluated. The antimicrobial effect of neutralized cell-free supernatants (nCFS) was assessed in vitro on polystyrene microtiter plates. Furthermore, molecular and morphological analyses were executed to evaluate the production of bioactive substances. To determine the nature of antimicrobial substance present in nCFS of *B. licheniformis* A-1-5B-AP, nCFS was exposed to the activity of various enzymes. The nCFS of *B. licheniformis* A-1-5B-AP significantly (*p* < 0.0001) reduced the growth of *Porphyromonas gulae* 3/H, *Prevotella intermedia* 1/P and *Streptococcus mutans* ATCC 35668. On the other hand, *B. licheniformis* A-2-11B-AP only significantly (*p* < 0.0001) inhibited the growth of *P. intermedia* 1/P and *S. mutans* ATCC 35668. However, enzyme-treated nCFS of *B. licheniformis* A-1-5B-AP did not lose its antimicrobial effect and significantly (*p* < 0.0001) inhibited the growth of *Micrococcus luteus* DSM 1790. Further studies are needed for the identification of antimicrobial substances.

## 1. Introduction

In recent years, the use of probiotic bacteria and their bioactive substances to inhibit the growth of oral pathogenic bacteria has gained a growing interest [1,2,3]. So far, most of the investigated probiotic bacteria have been members of intestinal microbiota [2], with emphasis on *Lactobacillus* and *Bifidobacterium* being the main studied genera [4,5]. However, it is assumed that probiotics of oral origin would be more effective for adapting to the surfaces of the oral cavity [6].

Probiotic strains belonging to the genus *Bacillus* have shown to be transient colonizers of the host. Both forms, vegetative and spore, have been used as probiotics. Their antimicrobial effect is a result of the production of different metabolites, such as bacteriocins, biosurfactants (BS) and exopolysaccharides (EPS) [4].

*Bacillus licheniformis* is a Gram-positive, endospore-producing bacterium that belongs to the *Bacillus subtilis* group [7,8]. Bacteria of this group are considered to be relatively safe, but certain strains can cause opportunistic infections [9]. In comparison to most bacilli, which are in general aerobic, *B. licheniformis* is a facultative anaerobe [10] and is commonly found in natural environments such as soil or plants [11,12]. Some strains or their bioactive substances have been included in probiotics for human and veterinary use, and the aquaculture, biomedicine, pharmaceutical and food industries or in environmental applications [13,14]. *B. licheniformis* could promote animal health by stimulating the immune system, enhancing function of mucosal barriers, inhibiting the colonization of pathogenic bacteria, promoting the proliferation of potentially beneficial microorganisms and maintaining the balance of microbiota [15].

*B. licheniformis* is able to produce a wide range of antimicrobial substances with inhibitory activity against a broad spectrum of bacteria [9,13], and due to the production of these extracellular substances, *B. licheniformis* is considered to be an economically interesting microorganism. For example, it is used for the manufacturing of antibiotics and enzymes such as amylases or proteases [8,10,11]. Bacitracin is the first peptide antibiotic derived from cultures of *B. licheniformis* and has been applied widely in the medical and veterinary area with excellent safety. This antibiotic is a mixture of at least 5 polypeptides, and consists of 3 separate compounds, bacitracin A, B and C. It is active against various species of Gram-positive and a few species of Gram-negative bacteria [16,17]. In addition to that, some strains can produce two-peptide lantibiotic bacteriocin lichenicidin [18]; however, synthesis of bacteriocin-like peptides such as lichenin and bacillocin was also reported [13]. Moreover, *B. licheniformis* can synthesize EPS of various biological activities, including antibacterial and antioxidant effects [19]. A typical example is levan [20], which is synthesized by an enzyme, levansucrase [21]. Inhibitory effects of *B. licheniformis* can also be associated with the non-ribosomal synthesis of lipopeptide BS lichenysin [22,23]. These biological properties make *B. licheniformis* a potential candidate for the development of new probiotics.

Therefore, the aim of present study was to investigate in the vitro inhibitory effect of *B. licheniformis* strains isolated from canine dental biofilm against canine and human oral pathogens.

## 2. Materials and Methods

### 2.1. Identification of Bacterial Strains

Bacterial strains *B. licheniformis* A-1-5B-AP and *B. licheniformis* A-2-11B-AP isolated from canine dental biofilms were cultured on blood agar (BA) at 37 °C for 24 h under aerobic conditions and identified based on the 16S rRNA gene and *gyrB* gene. BA was prepared as Tryptone Soya Agar (HiMedia, Mumbai, India) supplemented with 5% sterile horse blood. For identification by the 16S rRNA gene, DNA was extracted from pure bacterial cultures using DNAzol Direct (Molecular Research Center Inc., Cincinnati, USA) according to the manufacturer’s instructions. For identification by *gyrB* gene, DNA was isolated using Quick-DNA Fecal/Soil Microbe Miniprep Kit (Zymo Research, Irvine, USA). PCR reaction was performed in a thermocycler (TProfesional Basic, Biometra GmbH, Gôttingen, Germany) using OneTaq 2X Master Mix with standard buffer (New England Biolabs, Foster City, CA, USA) and universal primers (27F, 1492R) for the 16S rRNA gene [24] and Blich-F1: 5′AKACGGAAGTGACGGGAAC3′ and Blich-R1: 5′AGAAACTTTTCRAGCGCTT3′ for *gyrB* gene according to Huang et al. [25].

The PCR conditions for the 16S rRNA gene consisted of an initial denaturation at 94 °C for 5 min, followed by 30 cycles including denaturation at 94 °C for 1 min, annealing at 55 °C for 1 min and extension at 72 °C for 3 min and final extension at 72 °C for 10 min. The PCR conditions for *gyrB* gene were 5 min at 95 °C, 30× [1 min at 95 °C, 1 min at 65 °C and 1 min at 72 °C] and 5 min at 72 °C. *B. licheniformis* DSM 13 (German Collection of Microorganisms and Cell Cultures, Braunschweig, Germany) was used as a positive control. A negative control (RNase-free water) was included in the PCR reactions. The expected product size for the *gyrB* gene was 613 bp.

PCR products were visualized on a 2% agarose gel under UV light using GelRed [26] (Biotium, Inc., Hayward, CA, USA) and sent for purification and Sanger sequencing at Microsynth (Vienna, Austria). The obtained sequences were processed using Geneious 8.0.5 program (Biomatters, Auckland, New Zealand). Then, they were compared to the NCBI GenBank database using BLASTn [27]. The sequences of the 16S rRNA genes were stored in GenBank database under the accession numbers (AN) MT492074 and MT492090.

### 2.2. Preparation of Neutralized Cell-Free Supernatants

*B. licheniformis* A-1-5B-AP, A-2-11B-AP and DSM 13 were inoculated on Brain Heart Infusion (BHI) agar (HiMedia, Mumbai, India) and grown at 37 °C for 24 h under aerobic conditions. A standardized suspension of the individual strains was prepared by resuspending the solitary colonies in 3 mL of saline solution, and turbidity was then adjusted to 1–1.1 McFarland at 565 nm wavelength (DEN-1 McFarland densitometer, Biosan, Riga, Latvia). After that, 0.5 mL suspension was inoculated into 50 mL BHI broth (HiMedia, Mumbai, India) and incubated on a shaker aerobically for 24 h at 37 °C and 119 rpm (SKO-D XL, Agrolab, Pischelsdorf, Austria). BHI broth inoculated with saline solution was used as a control. Subsequently, the inoculated BHI broth were centrifuged for 40 min at 4 °C and 4500 rpm (ROTINA 420R, Hettich, Tuttlingen, Germany). The obtained cell-free supernatant (CFS) was neutralized with 10 M NaOH to pH 7 and filtered through a microbiological filter with a pore size of 0.22 μm (Minasart; Biotech, Göttingen, Germany).

### 2.3. Antimicrobial Effect of B. licheniformis Strains

The oral pathogenic strains *Porphyromonas gulae* 3/H, *Prevotella intermedia* 1/P and *Streptococcus mutans* ATCC 35668 were used to test the antimicrobial effect of neutralized CFS (nCFS) of *B. licheniformis* strains. *P. gulae* 3/H and *P. intermedia* 1/P (periodontal disease associated bacteria) were isolated from canine dental biofilm and grown on BA at 37 °C for 72 h under anaerobic conditions (BBL GasPakTM Plus, Becton, Dickinson and Co., Franklin Lakes, NJ, USA). *S. mutans* was selected as human oral pathogen causing dental caries. *S. mutans* ATCC 35668 was acquired from the Faculty of Natural Sciences of Comenius University in Bratislava. It was inoculated on BA plates and incubated aerobically for 24 h at 37 °C.

For the testing of canine oral pathogens, 110 µL of a standardized suspension of pathogenic strains *P. gulae* 3/H and *P. intermedia* 1/P (1–1.1 McFarland) were pipetted into 11 mL tubes, and the tubes were filled to 11 mL with individual nCFS enriched with yeast extract (5 g/L; Condalab, Madrid, Spain), cysteine (1 g/L; Sigma-Aldrich, St. Louis, MO, USA), hemin (5 mg/L; Sigma-Aldrich, St. Louis, MO, USA) and vitamin K1 (1mg/L; Sigma-Aldrich, St. Louis, MO, USA). BHI broth was enriched with yeast extract before autoclaving, while cysteine, hemin and vitamin K1 were filtered and added afterwards. Individual enriched nCFS with saline solution were used as a negative control. An enriched BHI broth with pathogenic strains without nCFS was used as a positive control. To prevent oxygen access, the tubes were parafilm-coated and cultured for 72 h at 37 °C. After cultivation, the volume of each tube was homogenized and pipetted into the wells of a polystyrene microtiter plate (Greiner Bio-One GmbH, Frickenhausen, Germany) at 200 µL per well.

For human oral pathogen *S. mutans* ATCC 35668, individual nCFS were pipetted into the wells of a polystyrene microtiter plate at 180 µL per well. Subsequently, 20 µL of a standardized suspension of *S. mutans* ATCC 35668 (1–1.1 McFarland) were pipetted into the medium and nCFS. BHI broth and individual nCFS with saline solution were used as a negative control. BHI broth with *S. mutans* ATCC 35668 without nCFS was used as a positive control. Microtiter plates were cultured for 24 h at 37 °C.

To evaluate the growth of pathogenic strains in the presence of individual nCFS, absorbance at 570 nm was measured (Synergy 4 Multi-Mode Microplate Reader, BioTek, Winnoski, VT, USA). Individual strains were tested in at least three independent experiments and the results are interpreted as the arithmetic mean of the measured values ± the standard deviation.

### 2.4. Bacitracin Susceptibility Test of Oral Pathogens

Disk diffusion test was used to evaluate the susceptibility of *Micrococcus luteus* DSM 1790 and oral pathogens, namely *P. intermedia* 1/P and *S. mutans* ATCC 35668 to bacitracin. *M. luteus* DSM 1790 was purchased from Leibniz Institute DSMZ (German Collection of Microorganisms and Cell Cultures, Braunschweig, Germany). Turbidity of standardized suspension of individual bacterial strains was adjusted to 0.5 McFarland and inoculated onto BA plate [28]. A disk containing 10 units of bacitracin B (HiMedia, Mumbai, India) was placed on top of the inoculated agar plate, which was then incubated at 37 °C for 24, 48 and 72 h in case of *M. luteus* DSM 1790, *S. mutans* ATCC 35668 and *P. intermedia* 1/P, respectively. *P. intermedia* 1/P was cultivated under anaerobic conditions. After incubation, the inoculated plates were examined for inhibition zones. Growth inhibition zone diameter was measured in millimeters for all bacterial strains.

### 2.5. Evaluation of the Ability to Produce Bioactive Substances

The ability to produce bioactive substances was assessed based on molecular and morphological analyses. Production of EPS was evaluated phenotypically. *B. licheniformis* strains were inoculated on modified BHI agar with high sucrose content (100 g/L) and cultured aerobically at 37 °C for 24 h. The phenotypic manifestation of EPS production was assessed visually by forming viscous fiber or mucoid growth called as “ropy” and “nonropy” manifestation, respectively. A screening test was used to monitor the production of BS in *B. licheniformis* strains. The oil spreading test was performed according to Morikawa et al. [29]. Firstly, 20 mL of distilled water were added to the Petri dish and 20 μL of crude oil was dripped onto the water surface, followed by 10 μL of nCFS of tested strains. BHI broth without surfactant was used as a negative control and Tween 80 was used as a positive control.

*B. licheniformis* strains were tested by PCR for the presence of *lsRN* gene encoding the enzyme levansucrase, which is involved in the synthesis of levan EPS; the *bli04127* gene, which represents the structural gene of *Bliα* lichenicidin peptide; and *lchAA* gene encoding lichenysin synthetase. DNA isolated by Quick-DNA Fecal/Soil Microbe Miniprep Kit, OneTaq 2X Master Mix with standard buffer, RNase-free water and primers were used for PCR. Primers and PCR reaction conditions are listed in Table 1. *B. licheniformis* DSM 13 was used as a positive control and RNase-free water was used as a negative control. PCR products were visualized on a 2% agarose gel under UV light using GelRed and submitted for purification and Sanger sequencing to Microsynth. The obtained sequences were processed using the Geneious program. Subsequently, they were compared to the NCBI GenBank database using BLASTn. The sequences of the *lsRN*, *bli04127* and *lchAA* genes were stored in GenBank database under AN ON081292, ON081293, ON649684 and ON649685.

### 2.6. Effect of Enzymes on Antimicrobial Activity

To evaluate the effect of enzymes on antimicrobial substances, nCFS of *B. licheniformis* A-1-5B-AP were treated with proteinase K (1 mg/mL), lipase (1 mg/mL) and α-amylase (1 mg/mL). The enzymes were activated by incubating the enzyme-treated nCFS at 37 °C for 2 h, and then the enzymes were immediately inactivated at 95 °C for 5 min. Individual enzyme-treated nCFS were pipetted into the wells of a polystyrene microtiter plate at 180 µL per well. Subsequently, 20 µL of a standardized suspension of *M. luteus* DSM 1790 (1–1.1 McFarland) was pipetted into the medium. Enzyme-treated BHI broth with saline solution and individual enzyme-treated nCFS with saline solution were used as a negative control. BHI broth treated with enzymes with *M. luteus* DSM 1790 without nCFS was used as a positive control. Microtiter plates were cultured at 37 °C for 24 h. Subsequently, the absorbance at 570 nm was measured. The effect of the enzymes was tested in at least three independent experiments and the results are interpreted as the arithmetic mean of the measured values ± the standard deviation.

### 2.7. Statistical Analyses

Antimicrobial activity results were evaluated by one-way analysis of variance (ANOVA) with an additional Dunnett’s test in the GraphPad Prism 9.3.1 (GraphPad Inc., San Diego, CA, USA). The growth of *M. luteus* DSM 1790 in the presence of enzyme-treated nCFS of *B. licheniformis* A-1-5B-AP was evaluated by unpaired *t* test. A *p*-value less than 0.05 (*p* < 0.05) was considered statistically significant. The antimicrobial activity of our strains *B. licheniformis* A-1-5B-AP and A-2-11B-AP against selected pathogenic bacteria was compared with the antimicrobial activity of *B. licheniformis* DSM 13.

## 3. Results

### 3.1. Identification of Bacterial Strains

Based on BLASTn analysis for 16S rRNA gene, bacterial isolates A-1-5B-AP and A-2-11B-AP showed high similarity of 99.59% and 99.65%, respectively, with multiple *B. licheniformis* strains including *B. licheniformis* DSM 13 (AN: NR118996). PCR reaction with specific primers for *gyrB* gene was used to confirm the identification of *B*. *licheniformis* strains. A PCR product of approximately 600 bp was present in both tested strains and the positive control (*B. licheniformis* DSM 13). BLASTn analysis confirmed the initial identification.

*B. licheniformis* A-1-5B-AP had 99.64% similarity with three DNA gyrase subunit B (*gyrB*) genes of *B. licheniformis* strains, namely *B. licheniformis* UTM118 (AN: KF952583), *B. licheniformis* UTM102 (AN: KF952576) and *B. licheniformis* C32 (AN: HQ336651). *B. licheniformis* A-2-11B-AP had 100% similarity with multiple complete genomes and multiple *gyrB* genes of *B. licheniformis* strains.

### 3.2. Antimicrobial Effect of B. licheniformis Strains

**Figure 1:**
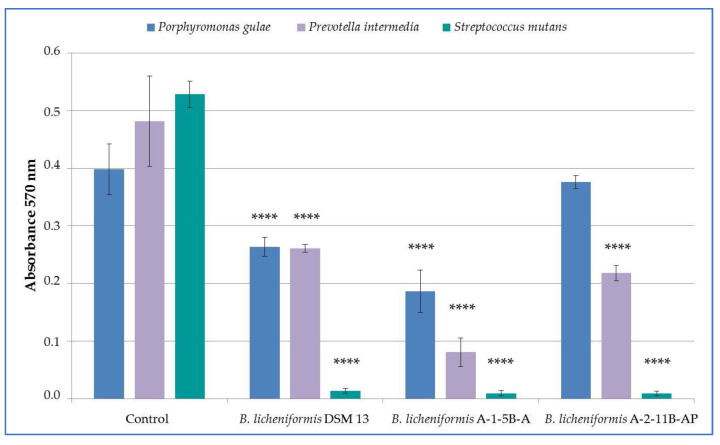
Inhibitory activity of nCFS of *Bacillus licheniformis* strains against the growth of oral pathogens; data are presented as the arithmetic means ± standard deviation; **** (*p* < 0.0001)—significant difference compared to the non-treated control.

Oral pathogenic strains were used to test the antimicrobial effect of *B. licheniformis* DSM 13, A-1-5B-AP and A-2-11B-AP. All tested nCFS of *B. licheniformis* strains had an inhibitory effect on the growth of pathogenic strains of *P. gulae* 3/H, *P. intermedia* 1/P and *S. mutans* ATCC 35668 (Figure 1). The growth of all pathogens tested was significantly inhibited (*p* < 0.0001) in nCFS of *B. licheniformis* DSM 13 and A-1-5B-AP compared to control. nCFS of *B. licheniformis* A-2-11B-AP significantly inhibited the growth of *P*. *intermedia* 1/P and *S. mutans* ATCC 35668 (*p* < 0.0001) but did not have a significant inhibitory effect on the growth of *P. gulae* 3/H. In terms of evaluating the percentage of growth inhibition, in *P. gulae* 3/H it ranged from 5.57 to 53.25%, in *P. intermedia* 1/P from 45.78 to 83.29%, and in *S. mutans* ATCC 35668 it was above 97% for all strains tested (Table 2).

### 3.3. Bacitracin Susceptibility Test

In order to exclude the possible inhibitory effect of bacitracin in the nCFS of *B. licheniformis* strains, pathogenic strains were tested for bacitracin susceptibility. In *S. mutans* ATCC 35668, a small inhibition zone diameter of 11 mm was present after 24 h incubation. On the other hand, the size of inhibition zone for *P. intermedia* 1/P was 63 mm. The diameter of the inhibition zone for *M. luteus* DSM 1790 was 28 mm, thus concluding that sensitivity to the bacitracin of *S. mutans* ATCC 35668 is intermediate, while *P. intermedia* 1/P and *M. luteus* DSM 1790 are sensitive. 

### 3.4. Production of Bioactive Substances

**Figure 2:**
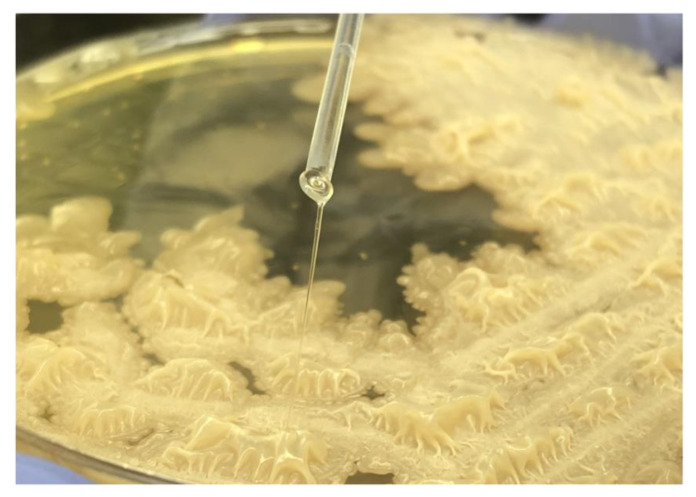
Forming of viscous filament known as ropy phenotype in *B. licheniformis* A-1-5B-AP.

The manifestation of EPS production was observed as the qualitative assessment of the phenotypic manifestation in both tested strains of *B. licheniformis*. *B. licheniformis* A-1-5B-AP formed a viscous fiber, also known as the “ropy” phenotype (Figure 2). *B. licheniformis* A-2-11B-AP had a typical mucoid growth with the “non-ropy” phenotype with shiny colonies that are characteristic for EPS production on BHI agar supplemented with 10% sucrose.

The oil spreading test was used to detect the production of BS in *B. licheniformis* strains. Tween 80 was used as a positive control and it created an oil-free clearing zone when dropped on the surface of crude oil. However, both tested strains of *B. licheniformis* and the negative control showed no clearing zone associated with surfactant activity.

PCR reaction with specific primers was used for the detection of the *bli04127* gene responsible for the synthesis of one antimicrobial peptide of the two-peptide lantibiotic lichenicidin. Based on the agarose gel electrophoresis, the PCR products had sizes of approximately 215 bp in both the positive control and *B. licheniformis* A-2-11B-AP. No PCR products were present in the negative control and *B. licheniformis* A-1-5B-AP. Following Sanger sequencing, sequences of *B. licheniformis* A-2-11B-AP and lichenicidin-producing strain *B. licheniformis* DSM 13 that was used as a positive control showed 100% homology. Based on BLASTn analysis, *B. licheniformis* A-2-11B-AP showed 100% homology with several complete *B. licheniformis* genomes, including *B. licheniformis* DSM 13 (AN: AE017333) and with the lantibiotic gene cluster of *B. licheniformis* VK21 (AN: GU949560).

*B. licheniformis* strains are able to produce multiple EPS, including levan, with antibacterial and antibiofilm activity. Levan is synthesized by levansucrase enzyme encoded by the *lsRN* gene, which was detected by PCR with specific primers BlLs-F and BlLs-R. Regarding agarose gel electrophoresis PCR products of approximately 1800 bp were present in positive control and *B. licheniformis* A-2-11B-AP. However, no PCR products were present in *B. licheniformis* A-1-5B-AP and the negative control. Following Sanger sequencing, the sequences of the levan-producing strain *B. licheniformis* DSM 13 were used as positive control, and *B. licheniformis* A-2-11B-AP showed 100% homology. Based on BLASTn analysis, *B. licheniformis* A-2-11B-AP showed 100% homology with several complete *B. licheniformis* genomes, including *B. licheniformis* DSM 13 (AN: AE017333) and to the levansucrase gene of *B. licheniformis* 8-37-0-1 (AN: KF647836).

Most of the *B. licheniformis* strains are able to synthesize surface-active substances, including lichenysin with antibacterial and antibiofilm activity. PCR with the set of specific primers LicA-F and LicA-R was used for the detection of lichenysin synthetase gene. Using agarose gel electrophoresis, PCR products of 735 bp were present in all strains tested including the positive control. No PCR product was present in the negative control. Based on BLASTn analysis, *B. licheniformis* A-1-5B-AP showed 99.86% homology with the *B. licheniformis* lichenysin biosynthesis operon: the lichenysin synthetase A (*licA*), lichenysin synthetase B (*licB*), lichenysin synthetase C (*licC*), and thioesterase (*licTE*) genes, complete cds (AN: U95370). *B. licheniformis* A-2-11B-AP showed 100% similarity with multiple complete *B. licheniformis* genomes and 91.77% identity with the *B. licheniformis* lichenysin biosynthesis operon: lichenysin synthetase A (*licA*), lichenysin synthetase B (*licB*), lichenysin synthetase C (*licC*) and thioesterase (*licTE*) genes, complete cds (AN: U95370).

### 3.5. Effect of Enzymes on Antimicrobial Activity of nCFS B. licheniformis A-1-5B-AP

**Figure 3:**
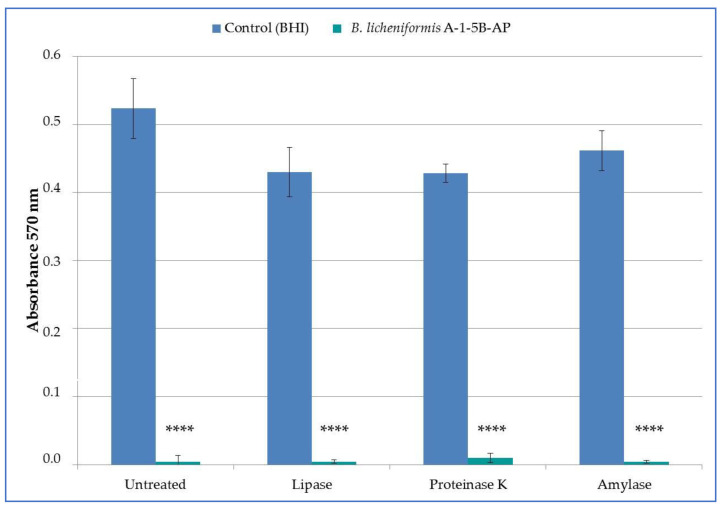
Enzymatic treatment of neutralized cell-free supernatants of *B. licheniformis* A-1-5B-AP and its activity against indicator strain of *Micrococcus luteus* DSM 1790; data are presented as the arithmetic means ± standard deviation; **** *p* < 0.0001—significant difference compared to the non-treated control.

To determine the nature of antimicrobial substance synthesized by *B. licheniformis* A-1-5B-AP, nCFS was treated with various enzymes. However, presence of neither enzyme (proteinase K, lipase and α-amylase) affected the antimicrobial activity of nCFS and addition of these enzymes was not accompanied with the loss of the antimicrobial activity of nCFS (Figure 3). All enzyme-treated nCFS significantly (*p* < 0.0001) inhibited the growth of *M. luteus* DSM 1790. The percentage of growth inhibition was 99.16 ± 0.48, 98.17 ± 1.33, and 99.22 ± 0.41% for lipase, proteinase K, and α-amylase, respectively.

## 4. Discussion

Oral bacteria associated with periodontal diseases and dental caries are receiving considerable attention in order to evaluate the antimicrobial effect of natural substances that could positively affect oral health [34]. To the best of our knowledge, there are currently no studies available describing the inhibitory effect of canine oral strains of *B. licheniformis* against canine and human oral pathogens. Therefore, the present study evaluated the inhibitory effect of nCFS of *B. licheniformis* A-1-5B-AP and A-2-11B-AP isolated from canine dental biofilms against selected oral pathogenic strains, namely *P. gulae* 3/H, *P. intermedia* 1/P and *S. mutans* ATCC 35668.

In general, *P. gulae* and *P. intermedia* are associated with periodontal disease in dogs [35], while *S. mutans* plays a major role in the development of dental caries in humans and also promotes formation of oral biofilms [36]. In regard to the periodontal diseases, there is only limited amount of knowledge available about the effectiveness of *Bacillus* spp. [37], even though members of the genus *Bacillus* are considered to be relatively good producers of antimicrobial substances. In particular, *B. licheniformis* has been described as a source of many antimicrobial substances [13]. A mouthwash containing *Bacillus subtilis* has shown efficacy in reducing periodontal pathogens in humans [38]. In experimental periodontitis in rats, the beneficial effects and possible therapeutic potential of *B. subtilis* and *B. licheniformis* were also described [37]. In this study, nCFS of *B. licheniformis* A-1-5B-AP and DSM 13 strains showed an antimicrobial effect against *P. gulae* 3/H and *P. intermedia* 1/P, while nCFS of *B. licheniformis* A-2-11B-AP significantly inhibited only the growth of *P. intermedia* 1/P.

Dental caries is considered to be one of the most common oral diseases in human population with a relatively high prevalence [39]. It has been shown that some enzymes and antimicrobial compounds produced by some *Bacillus* spp. inhibit the growth of *S. mutans*, thereby preventing biofilm formation [36]. *Bacillus coagulans* in chewable tablet was effective in reducing and inhibiting caries-causing mutans streptococci and lactobacilli levels in plaque and saliva in children [40]. On the other hand, in the study by Rivis et al. [41], the inhibitory effect of the combination of *B*. *subtilis* B-5007 and *B. licheniformis* B-5514 on the growth of the clinical strain *S. mutans* was not demonstrated. Compared to humans, the occurrence of dental caries in dogs is rare, probably due to the key factors such as differences in the oral microbiota composition, higher pH of saliva or the association with a low-carbohydrate diet. From this point of view, it is assumed that canine oral bacteria could be applied in the control of *S. mutans* and other cariogenic bacteria in human dental biofilms [42]. In this study, all tested nCFS of *B. licheniformis* significantly inhibited the growth of *S. mutans* ATCC 35668, indicating the ability to produce antimicrobial substances. Similarly, in the study by Martins et al. [42], *Bacillus* sp. isolated from canine dental biofilm showed bacteriocinogenic activity against *S. mutans*.

Since some strains of *B. licheniformis* are able to produce bacitracin, a sensitivity test of oral pathogens to bacitracin was also performed. Bacitracin is a non-ribosomally synthesized docapeptide antibiotic produced by certain strains of *B. subtilis* and *B. licheniformis* [43]. It has activity mainly against Gram-positive and some Gram-negative bacteria by inhibiting bacterial cell wall biosynthesis [44], and it can also inhibit biofilm formation in cariogenic *S. mutans* [45]. In the studies of Anumala et al. [46], up to 80% of *P. intermedia* strains showed sensitivity to bacitracin. In addition, the inhibition of growth and biofilm formation of *M. luteus* by bacitracin has been demonstrated [47]. Bacitracin B susceptibility testing performed in this study showed that *P. intermedia* 1/P and *M. luteus* DSM 1790 strains were also susceptible, which is in agreement with previous claims. However, *S. mutans* ATCC 35668 showed intermediate sensitivity to bacitracin B, which is in contrast to the result in the study by Kazemi et al. [48], in which the same strain was resistant to 15 μg of bacitracin contained in a disc. It is generally known that *S. mutans* is resistant to bacitracin, or it contains bacitracin resistance genes. This ability is often used to isolate *S. mutans* from the entire spectrum of oral microbiota using media containing bacitracin [49,50].

*Bacillus* spp. are prolific in the production of antimicrobial substances which may give them a selective advantage over other bacterial species [51]. Levan is a fructan-type EPS produced from sucrose via extracellular levansucrases by many bacteria, including *B. licheniformis*, and its synthesis can occur both under aerobic and anaerobic conditions [52,53]. Levans are involved in many physiological functions, such as the attachment of bacterial cells in their habitat and protection from cold and drought. In addition, levans have a high potential as substances with antibacterial, antibiofilm, antiviral and anticarcinogenic effects [30,54]. BS are amphiphilic compounds produced by microorganisms with significant surface and emulsifying activities [55]. A surface active substance known as lichenysin is produced by *B. licheniformis* as a secondary metabolite, and its biosynthesis is catalyzed by non-ribosomal peptide synthetases. Its structure is very similar to that of surfactin, a well-known BS produced by *B. subtilis*. Both compounds can be produced under aerobic or anaerobic conditions [7]. In this study, EPS production was demonstrated phenotypically in both tested strains of *B. licheniformis*, while in *B. licheniformis* A-2-11B-AP, a gene encoding levansucrase synthesis was detected by molecular analysis as well. The lichenysin synthetase gene was present in all strains, but the oil spreading test did not demonstrate the presence of this substance in nCFS. Even though the presence of lichenysin synthetase genes in *B. licheniformis* seems to be very frequent, their production is closely related to environmental conditions, such as the incubation temperature or the type of carbon, nitrogen and phosphate sources present in the culture medium [7].

In our tested strains, the ability to produce lichenicidin was demonstrated only in *B. licheniformis* A-2-11B-AP; however, its nCFS did not significantly inhibit the growth of *P. gulae* 3/H compared to nCFS of *B. licheniformis* A-1-5B-AP. Lichenicidin is a class II dipeptide lantibiotic that was first described in *B. licheniformis* DSM 13 [56]. Moreover, it has been shown that lichenicidin can be produced by other strains of *B. licheniformis*, and the structure of its peptides may differ depending on the producing strain [57]. Furthermore, the production of several bacteriocin-like substances with different characteristics and a wide spectrum of activity against pathogenic bacteria, such as lichenin, bacillocin 490 and P40, was also recorded in *B. licheniformis* strains [58,59].

In contrast to the lichenicidin-producing strains, *B. licheniformis* A-1-5B-AP showed higher inhibitory activity against all oral pathogens, indicating that this strain is capable of producing a different antimicrobial substance. To elucidate the nature of this antimicrobial substance, nCFS of *B. licheniformis* A-1-5B-AP was treated with different enzymes, namely proteinase K, lipase and α-amylase, and tested against the indicator strain of *M. luteus* DSM 1790. However, proteinase K, lipase or α-amylase did not affect the antimicrobial activity present in the nCFS of this strain. Many antimicrobial peptides produced by *Bacillus* spp. have different resistance to enzyme activity, with stability over a wide range of pH and temperature [60]. The bacteriocin lichenocin 50.2 is completely inactivated by proteinase K and pronase E, and partial inactivation is observed upon treatment with trypsin. BLIS_SXAU06 produced by *B. licheniformis* SXAU06 can be fully inactivated by pronase E and partially inactivated by trypsin and pepsin, but it is insensitive to proteinase K [61]. Antimicrobial activity of a bacteriocin-like substance JY-1 present in the supernatant of *Bacillus* spp. JY-1 was not affected by neutral proteinase and proteinase K, but it was partially reduced when treated with trypsin and pepsin [62]. The effect of enzymes including pepsin, trypsin and proteinase K was tested on CFS of *B. licheniformis* MCC2514 and MCC2512 against *M. luteus* ATCC9341, and while the antimicrobial compounds found in the supernatants were sensitive to proteinase K, they were resistant to the other protease enzymes tested. Supernatants of these strains were also treated with α-amylase, and showed 50% residual activity [63]. In addition, the possible production of bacitracin could inhibit proteinase K [64] as it is known to be capable of protease inhibition [65].

## 5. Conclusions

The growth of *P. gulae* 3/H, *P*. *intermedia* 1/P, *S. mutans* ATCC 35668 and *M. luteus* DSM 1790 was significantly inhibited by nCFS of *B. licheniformis* A-1-5B-AP. Based on molecular analysis, the presence of genes associated with the synthesis of lichenysin was detected, although its presence in medium was not confirmed. Furthermore, *B. licheniformis* A-1-5B-AP was able to produce EPS, which was manifested by the formation of viscous fiber known as the ropy phenotype. Therefore, to determine the nature of the antimicrobial substance produced by *B. licheniformis* A-1-5B-AP, nCFS was treated with various enzymes. However, there was no loss of antimicrobial activity detected after treatment. Due to the inhibitory activity of *B. licheniformis* A-1-5B-AP, it has a potential for possible use in the prevention and elimination of not only periodontal pathogens in dogs but also cariogenic *S. mutans* in humans. Further studies are needed to clarify the identity and characterize the antimicrobial substance present in nCFS of *B. licheniformis* A-1-5B-AP.

## Figures and Tables

**Table 1:** Primers and PCR conditions.

Target Sequence (Gene)	Primer Sequence (5′ to 3′)	PCR Conditions	Product Size (bp)	Source
Levansucrase (*lsRN*)	TGCTCTAGACGATTCCCGCTTATACAGACTATAGAT	95 °C 3 min, 24× [95 °C 1 min, 55 °C 30 s, 72 °C 4 min] 72 °C 10 min	1793	[30]
	CGGGATCCTTATTTGTTTACCGTTAGTTCTCCC			
Lichenicidin (*bli04127*)	GGAAATGATTCTTTCATGG	95 °C 5 min, 30× [95 °C 1 min, 55 °C 1 min, 72 °C 1 min] 72 °C 5 min	215	[31,32]
	TTAGTTACAGCTTGGCATG			
Lichenysin synthetase (*lchAA*)	GTGCCTGATGTAACGAATG	94 °C 2 min, 30× [94 °C 15 s, 60 °C 30 s, 72 °C 50 s] 72 °C 5 min	735	[33]
	CACTTCCTGCCATATACC			

**Table 2:** Inhibition of oral pathogens growth by nCFS of *Bacillus licheniformis* strains.

	Percentage of Growth Inhibition (%)		
Bacterial Strains	*B. licheniformis* DSM 13	*B. licheniformis* A-1-5B-AP	*B. licheniformis* A-2-11B-AP
*P. gulae* 3/H	33.86 ± 4.12	53.25 ± 9.21	5.57 ± 2.78
*P. intermedia* 1/P	45.78 ± 1.38	83.29 ± 5.12	54.76 ± 2.78
*S. mutans* ATCC 35668	97.48 ± 0.79	98.24 ± 0.82	98.30 ± 0.79

## Data Availability

Not applicable.
